# Dynamic effects of smoking cessation on disease incidence, mortality and quality of life: The role of time since cessation

**DOI:** 10.1186/1478-7547-6-1

**Published:** 2008-01-11

**Authors:** Rudolf T Hoogenveen, Pieter HM van Baal, Hendriek C Boshuizen, Talitha L Feenstra

**Affiliations:** 1 National Institute for Public Health and the Environment, Bilthoven, The Netherlands

## Abstract

**Background:**

To support health policy makers in setting priorities, quantifying the potential effects of tobacco control on the burden of disease is useful. However, smoking is related to a variety of diseases and the dynamic effects of smoking cessation on the incidence of these diseases differ. Furthermore, many people who quit smoking relapse, most of them within a relatively short period.

**Methods:**

In this paper, a method is presented for calculating the effects of smoking cessation interventions on disease incidence that allows to deal with relapse and the effect of time since quitting. A simulation model is described that links smoking to the incidence of 14 smoking related diseases. To demonstrate the model, health effects are estimated of two interventions in which part of current smokers in the Netherlands quits smoking.

To illustrate the advantages of the model its results are compared with those of two simpler versions of the model. In one version we assumed no relapse after quitting and equal incidence rates for all former smokers. In the second version, incidence rates depend on time since cessation, but we assumed still no relapse after quitting.

**Results:**

Not taking into account time since smoking cessation on disease incidence rates results in biased estimates of the effects of interventions. The immediate public health effects are overestimated, since the health risk of quitters immediately drops to the mean level of all former smokers. However, the long-term public health effects are underestimated since after longer periods of time the effects of past smoking disappear and so surviving quitters start to resemble never smokers. On balance, total health gains of smoking cessation are underestimated if one does not account for the effect of time since cessation on disease incidence rates. Not taking into account relapse of quitters overestimates health gains substantially.

**Conclusion:**

The results show that simulation models are sensitive to assumptions made in specifying the model. The model should be specified carefully in accordance with the questions it is supposed to answer. If the aim of the model is to estimate effects of smoking cessation interventions on mortality and morbidity, one should include relapse of quitters and dependency on time since cessation of incidence rates of smoking-related chronic diseases. A drawback of such models is that data requirements are extensive.

## Background

Smoking is a risk factor for many major chronic diseases and reduces both length and quality of life [1]. In the Netherlands, about 28% of the Dutch population aged 15 or above smokes [2]. About 13% of the Dutch burden of disease and 3.7% of Dutch health care costs in 2003 could be attributed to smoking [3]. An effective tobacco control policy could thus substantially reduce the burden of disease. However, most of the effects of smoking on morbidity and mortality become manifest only after many years and not in all smokers. Since randomized trials to investigate the effect of smoking cessation interventions on disease occurrence and mortality are practically impossible, models based on current epidemiological knowledge and synthesizing data from many different sources are needed to estimate the effects of smoking cessation over time [4].

It was our aim to quantify the effects of smoking interventions on public health, taking into account the time since cessation of quitters. The latter is important, since many quitters relapse [5] and for most smoking related diseases the increased risks of former smokers only decrease gradually over time since cessation [6-8] Several models have been presented in the literature that deal with relapse and that include mortality risks of former smokers declining with time since quitting [9-13]. However, the potential use of these models is limited because they do not include all characteristics that are necessary to calculate the impact of smoking cessation on summary measures of population health combining morbidity and mortality, such as quality or disability adjusted life years. The SimSmoke model, for example, describes only all cause mortality and mortality from lung cancer [11,12]. The effects of changing smoking class prevalence rates on other chronic diseases is calculated afterwards at each time step.

In this paper we present a model that is capable of describing the effects of smoking cessation on morbidity and mortality over time, and that overcomes the limitations of the models mentioned above. Our model describes the life course of quitters after smoking cessation taking into account relapse. It distinguishes the most important smoking related chronic diseases, and the incidence rates of former smokers depend on time since cessation. Moreover, it explicitly describes morbidity from these diseases by modeling the change of the disease prevalence rates over time. Morbidity, in turn, determines mortality in the model. By modeling diseases, effects of smoking cessation on quality of life and health care costs can simply be estimated by coupling health care costs and quality of life figures to diseases [14] instead of coupling them directly to smoking status.

The model presented in this paper is part of the RIVM Chronic Disease Model (CDM) [15]. The CDM is a state-transition Markov-type model that was designed to describe the effects of epidemiological risk factors on morbidity and mortality from several chronic diseases in the Dutch population. It includes 28 chronic diseases and several risk factors amongst which smoking, Body Mass Index, and physical inactivity. In modeling diseases explicitly, the structure of the model is similar to the Prevent model [16] and the recently presented Quit Benefits model [10,17]. An important difference with the Prevent model is that also different risk factor classes are modeled. In comparison with the Quit Benefits Model our model includes more diseases, allows for comorbidity and has the ability to track health effects over a longer period. In the current study we describe how time since cessation is taken into account as an additional model parameter. In the next section, the model structure with respect to smoking is explained in detail. To illustrate the model, health effects are estimated of two interventions in which part of Dutch smokers quits smoking. To illustrate the strengths of the model its results are compared with the results of two simpler model versions. In the most simple version we assumed no relapse after quitting and equal incidence rates for all former smokers. To this were added incidence rates that depend on time since cessation. The final model was arrived at by also including relapse of quitters. In the discussion section, we will discuss the strengths and weaknesses of the model and elaborate on possible applications. Details on the mathematical structure of the model and its input data can be found in Appendices.

## Methodology

### Basic structure of the RIVM Chronic Disease Model (CDM)

The CDM is a model that describes the effects of risk factors, including smoking and overweight, on the incidence and mortality of chronic diseases in the population. It describes the effects for the total Dutch population taking into account birth and migration [15]. The CDM has been used for future projections of risk factor and disease prevalence numbers [18-21], cost effectiveness analyses [14] and estimates of healthy life expectancy [22]. The model describes the life course of cohorts in terms of transitions between risk factor classes and transitions between disease states over time. Risk factors and diseases are linked through relative risks of disease incidence. The CDM was formulated mathematically as a set of time-continuous differential equations [23]. The model equations describe the 1-year changes of the probability values for all risk factor classes and disease states in cohorts, specified by gender and age. The main model outcome variables are numbers of incident and prevalent cases and numbers of deaths, specified by disease, age and gender. To keep the number of model states manageable, the model describes the changes of the risk factor distributions and disease probabilities separately, i.e. as marginal distributions, but not the joint probability distribution function of all risk factor classes and diseases simultaneously.

The set of model equations consists of three components (see Appendix 1). In the initialization component the parameter values and the initial distribution of the population over all model states are calculated. In the simulation component the 1-year changes of the model state prevalence numbers are calculated. These changes are the result of transitions between the risk factor classes and disease states. The transition numbers are computed as the 1-year transition probabilities times the model state prevalence numbers at the start of the 1-year time-interval. Finally, in the post-processing component the values of the output variables are calculated from the results of the simulation component.

Demographic data such as all cause mortality rates and initial population numbers were available from Statistics Netherlands [24]. To estimate incidence, prevalence and mortality rates in the general population, three types of data sources were used: general practitioner registrations for non-cancer diseases, national cancer registries, and cohort studies for diabetes [25-27]. Non-cancer mortality rates were estimated using a three state transition model [28]. To compute health effects in terms of quality-adjusted life years (QALYs), the CDM couples disability weights from the Dutch Burden of Disease Study to disease prevalences [22]. Disability weights reflect the severity and impact of a disease relative to death and optimal health, defined as absence of disability, and ranges from 0 (no disability) to 1 (death). The Dutch Burden of Disease Study estimated disability weights for 48 different disease categories, using the person trade-off method. For the QALY calculations it was assumed that comorbidity reduces quality of life but that the effects are less than the sum of disability from the individual diseases[29,30]. Health care costs were calculated by coupling estimated disease prevalence numbers to costs per patient per year per disease available from the Dutch Costs of Illness study [14,31].

### Model structure with respect to smoking

The CDM relates smoking to increased incidence rates of 14 smoking-related chronic diseases, i.e. coronary heart disease (acute myocardial infarction (AMI) and other coronary heart disease), congestive heart failure, stroke, chronic obstructive pulmonary diseases (COPD), diabetes, and cancer of the lung, stomach, larynx, oral cavity, esophagus, pancreas, bladder and kidney. The incidence rates of smoking related diseases are increased in current smokers as well as in former smokers, with the relative risks of former smokers declining from the risk of a smoker immediately after stopping smoking to that of a never smoker as a function of time since cessation. Smoking status was defined as 'never smoker', 'current smoker', and 'former smoker'. The latter category was further subdivided into several classes based on the number of years since smoking cessation: less than 1 year ago, quitted between 1 and 2 years ago, and so forth, up to 20 years or more. The transitions involved were initiation (from 'never' to 'current' smoker), cessation (from 'current' to 'former, and quitted less than 1 year ago'), relapse (from any 'former' smoking class back to 'current'), and continuation as former smoker (e.g. from 'quitted between 2 and 3 years ago' to 'quitted between 3 and 4 years ago'). For all smoking-related diseases we distinguished two states, i.e. without and with the disease. For each disease the transition involved was 'disease incidence', i.e. from 'disease-free' to 'with the disease'. Finally, the model distinguishes outflow due to mortality from any cause. Mortality rates depend on the disease, i.e. persons with a disease have higher mortality rates than disease-free persons. The parameters used in the model are the 1-year probabilities of each transition between model states described.

In the next subsection, we describe in detail how the model parameters were estimated that depend on the time since cessation: relapse probabilities for former smokers and relative risks of smoking related diseases for former smokers.

### Input data and estimation of model parameters dependent on time since cessation

The initial distribution over all smoking classes and the 1-year smoking class transition probabilities were estimated from survey data from the Dutch Foundation on Smoking and Health [2]. This is a national survey on smoking which includes questions on current and past smoking status. Initiation and quit probabilities for never-smokers and current smokers were estimated using the retrospective data and are described elsewhere [32]. The initial smoking class probability values were calculated from the current smoking status. Since we had no input data on the initial distribution of all former smokers over time since smoking cessation, we generated this distribution by running the model initially for a birth cohort of non-smokers (see Appendix 1). The generated distribution was used as input data.

It was assumed that the relapse rates of quitters decrease over the time since smoking cessation, and that the decrease is according to a negative-exponential curve:

(1) *λ*(s) = *α β *exp(-*β*s)

with: 

s time since smoking cessation

*λ *smoking relapse rate

*α*, *β *regression coefficients.

The 1-year probability of relapse was calculated by applying this hazard rate to a 1-year time interval. The parameters *α *and *β *were estimated by fitting the probability of relapse to the data on the past history of former smokers [2,33]. Details and results of the estimation method are given in Appendix 2.

For the risk factor class 'current smoking' relative risks, specified by gender and age, were used (see the online appendix of [22]). The relative risks of former smokers gradually decrease over time since smoking cessation in the following way. Immediately after cessation the relative risks are similar to those of current smokers. In the long run and conditional on survival the negative health effects of smoking disappear, and the relative risks are similar to those of never smokers, i.e. they attain value 1. Therefore, it was assumed that for each disease the relative risk decreases according to a negative-exponential curve starting from the value for current smokers and converging to the value 1. Furthermore, it was assumed that the rate of convergence decreases with age. This was done to account for the cumulative nature of the effects of smoking. We estimated these relative risks using the following regression model:

(2) RR_former_(a,s) = 1 + (RR_current_(a) - 1) exp(- *γ*(a) s)

*γ*(a) = *γ*_0 _exp(-*η*a)

with: 

a age

s time since smoking cessation

*γ *regression coefficient of time dependency

*γ*_0_, *η *intercept and regression coefficient respectively of age dependency

RR_former_(a) relative risks of disease incidence for former smokers

RR_current_(a) relative risks of disease incidence for current smokers

The parameters *γ*_0 _and *η *were estimated in a two-step procedure, using relative risk data from major cohort studies reported in literature. In the first step we estimated the regression coefficient *γ*(a) for each study separately. Next, all regression coefficients *γ*(a) calculated were plotted in one graph against the mean age of the cohort. In the second step the regression coefficient *γ*_0 _and *η *were estimated from these *γ*(a) values. The details and results of the estimation method are given in Appendix 3.

## Results

To demonstrate our method as implemented in CDM, we calculated the health effects of two intervention scenarios in which part of Dutch current smokers in the Netherlands quits smoking:

- 'young quitters' scenario: 10% of all current smokers aged 20–44 quit smoking in year 1;

- 'old quitters' scenario: 10% of all current smokers aged 45–70 quit smoking in year 1.

To estimate the health effects of the intervention scenarios, the results were compared to a current practice scenario. In the current practice scenario, the CDM was run with the current distribution of smoking in the Netherlands as input and assuming that there would be no transitions between smoking classes over time. To show the effect of taking into account time since smoking cessation for former smokers, the estimated effects of the intervention scenarios were compared using three different versions of the model:

- 'simple' model: no relapse of quitters and relative risks of smoking related diseases do not depend on time since cessation.

- 'time dependent' model: relative risks of smoking related diseases depend on time since smoking cessation, but no relapse of quitters.

- 'time dependent & relapse' model: relapse of quitters and relative risks of smoking related diseases depend on time since smoking cessation.

**Figure 1:**
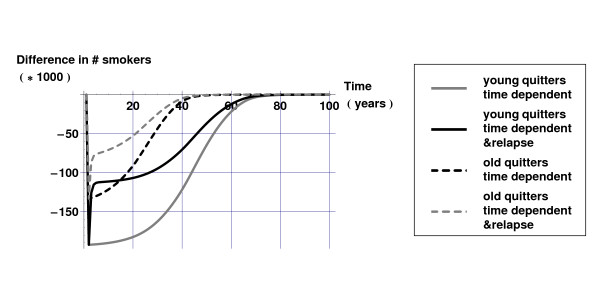
Differences in number of smokers for intervention scenarios compared to current practice scenario with 'time dependent' and 'time dependent & relapse' model versions.

Figure 1 displays the difference in the numbers of smokers for the 'young quitters' and 'old quitters' scenarios compared to the current practice scenario, both for the 'time dependent' and the 'time dependent & relapse' model. As can be seen from Figure 1, taking into account relapse influences the projections of the number of smokers considerably: in both scenarios approximately half of the extra quitters relapse within a year. However, after one year differences between the 'young quitters' and 'old quitters' scenarios emerge: in the scenario targeted at older smokers more of the extra quitters die.

**Figure 2:**
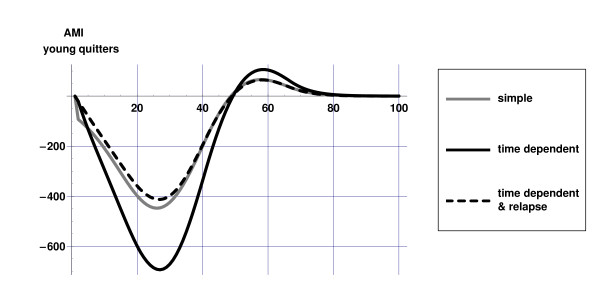
Difference in AMI incidence for three model versions in the 'young quitters' scenario compared to current practice scenario.

**Figure 3:**
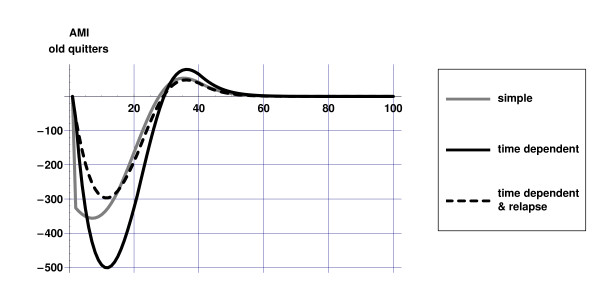
Difference in AMI incidence for three model versions in the 'old quitters' scenario compared to current practice scenario.

Figures 2 and 3 illustrate the differences between the dynamics of the 'simple' and 'time dependent' model: in both scenarios the initial decrease in AMI incidence is highest for the 'simple' model, since incidence rates decrease immediately after smoking cesssation. In the 'time dependent' models the gradual decrease of relative risks of former smokers becomes visible only after some time. After some time however, as relative risks in the 'time dependent' models approach those of never smokers, the effects on disease incidence become stronger in this model. This effect is more pronounced in the 'old quitters' scenario due to the fact that at higher ages disease incidence rates and relative risks are higher. Although the nature of the dynamics is similar as in the 'time dependent' model, the effects on disease incidence are less strong in the 'time dependent & relapse' model due to relapse.

**Figure 4:**
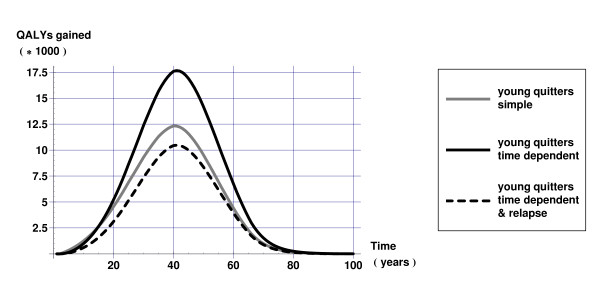
QALYs gained over time 'young quitters' scenario.

**Figure 5:**
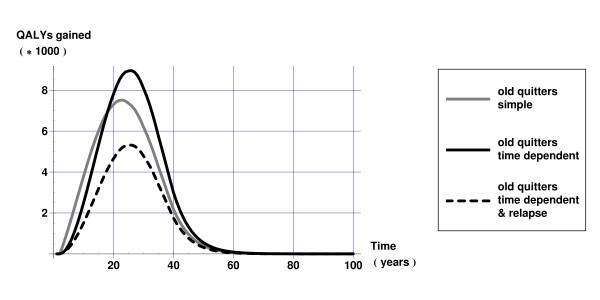
QALYs gained over time 'old quitters' scenario.

Figures 4 and 5 display QALYs gained over time calculated as the difference in QALYs between the intervention and current practice scenarios. Figure 4 reveals that health gains of smoking cessation are underestimated in younger populations if one does not account for time since cessation. Figure 5 shows more clearly that not taking into account that relative risks depend on duration of smoking cessation results in overestimating health gains in the short run but underestimating health gains in the long run. Both Figures 4 and 5 show that not taking into account relapse results in too optimistic estimates of the health gains of smoking cessation.

Table 1 displays cumulative health gains over a period of 100 years for the two intervention scenarios calculated with the three different model versions. Table 1 shows that in the end both in younger and older populations health gains are underestimated if one does not allow relative risks to depend on duration of cessation. This effect is more pronounced in younger populations. Furthermore, health gains are overestimated if relapse is not accounted for.

**Table 1:** Cumulative differences in (quality adjusted) life years between intervention and current practice scenarios over a period of 100 years

	**'young quitters' scenario**			**'old quitters' scenario**		
	**'simple' model**	**'time dependent' model**	**'time dependent & relapse' model**	**'simple' model**	**'time dependent' model**	**'time dependent & relapse' model**

**Life years gained** (* 1,000)	525	746	439	277	317	187
**QALYs gained** (* 1,000)	425	588	348	200	221	131

## Conclusion and Discussion

In this paper, we showed that not taking into account duration of smoking cessation time results in biased effect measures of interventions. The immediate public health effects are overestimated, since in reality quitters start with having the health risks of current smokers. The long-term public health effects are underestimated since after longer time periods the effects of past smoking have disappeared and so surviving quitters resemble more never smokers. On balance these two counteracting forces cause that health gains of smoking cessation are underestimated when one does not allow disease risks to depend on time since smoking cessation. This effect is more pronounced in younger populations.

These results show that simulation models are sensitive to assumptions regarding the model specification, in our case transition rates for former smokers. Therefore sensitivity analyses of simulation models should not only involve parameter values, but also model specifications should be taken into consideration. The model should be specified carefully to coincide with the questions it is supposed to answer. If the aim of the model is solely to project future health status in a business as usual scenario, a model without duration dependency might suffice. In the total population of former smokers, age correlates strongly with time since cessation. Therefore, using age specific relative risks without taking into account time since cessation would not introduce major errors if the aim is to make projections for the population of former smokers. Including time since smoking cessation becomes crucial when the aim of the modelling is to estimate effects of smoking cessation interventions instead of making projections.

A major drawback of the methodology employed is that more complex models require more detailed data, which are harder to get. For example, in the 'time dependent' models we used relative risks that were specified by both age and time since smoking cessation. The number of studies available to estimate these relative risks was much smaller than the number of studies available to estimate a single age-dependent relative risk value for all former smokers combined. The reduction of absolute health risks after smoking cessation depends both on the absolute and relative disease risks for smoking. Since the absolute risks are largest for elderly ages, the reductions are most sensitive to the relative risk values for these ages. This was illustrated, for example, by the discussion on the attribution of US mortality numbers to obesity, where the attributed number of deaths were initially overestimated by assuming age-constant relative risk values [34]. This was reason for us to analyze the effect of age on the reduction of health risks over time since smoking cessation. Indeed, empirical data (see Appendix 3) showed that the rate of decline decreased with age. As a result, the relative effects of smoking cessation are smaller for higher ages. Analogously, we analyzed the effect of age on the reduction of smoking relapse rates. However, the data available did not enable us to regress these rates on both time since cessation and age simultaneously. Moreover, these data were retrospectively obtained measurements, with the first measurement being the smoking status for the period 'more than 2 years ago'. As a result, we could do no better than assuming that the proportional reduction of the relapse rates was constant over the time since cessation and over age.

Including specific chronic diseases and relapse rates and relative risks that depend on time since smoking cessation makes the model presented well suited to calculate the impact of smoking interventions on morbidity and mortality results, when it is appropriate to take into account several effects simultaneously. Examples of these effects are dependency on age, dependency on time since smoking cessation, and competing morbidity and mortality risks. It also enabled us to calculate generic health measures such as disability-adjusted life years. The model calculates the difference in quality adjusted life years between smokers and never smokers, and hence shows the health gain that can be gained by policy measures. By using a disease specific approach, the causal effects on quality of life and health care costs can be estimated and used in cost effectiveness analysis.

## Appendix 1: Mathematical model equations

The model consists of three components. In the initialization component the parameter values and the initial distribution of the population over all model states are calculated from the input data. In the simulation component the 1-year changes of the model state prevalence numbers are calculated for each cohort separately. Each cohort is defined by its initial age value, i.e. a(0). These changes in the number of persons for each state are the result of transitions between the risk factor classes and between the disease states. The numbers of transitions are computed as the 1-year transition probabilities times the state prevalence numbers at the start of the 1-year time-interval. Finally, in the post-processing component the model output variables are calculated from the results of the simulation component. All model parameters and variables are specified by gender and age, but we omit the index for gender below for reasons of readability.

### Initialization component

The parameters calculated here are the 1-year baseline disease incidence rates, i.e. the rates of getting the disease for never smokers, and the mortality rates for other causes of death. The disease incidence rates for never smokers are calculated by dividing the overall incidence rates by the weighted sum of relative risks.

(3) $$i_{d,base}(a)=\frac{i_{d,0}(a)}{\sum_{c}RR_{d}(c,a)n_{c,0}(a)}$$

with: 

a age

c index over all smoking classes, c = never, current, former

i_d,0_(a) data disease d incidence rate at age a

i_d,base_(a) disease d incidence rate for never smokers at age a

n_c,0_(a) initial smoking class probability values at age a

RR_d_(c,a) relative risk of incidence of disease d for smoking class c

The relative risks for never and current smokers are input data (i.e. 'given'). The relative risks of former smokers are calculated (see Appendix 3). The other causes mortality rates describe the mortality rates for causes of death other than the smoking-related chronic diseases included in the model.

(4) $$\mu_{other}(a)=\mu_{tot}(a)-\sum_{d}am_{d}(a)p_{d,0}(a)$$

with: 

d index over diseases

*μ*_tot_(a) all cause mortality rates at age a

*μ*_other_(a) mortality rates for other causes of death at age a

p_d,0_(a) initial disease d prevalence rate values at age a

am_d_(a) disease d related attributable mortality rates at age a

The parameter am_d_(a) describes the mortality rates uniquely attributable to disease d. This parameter is defined as the additional mortality rate of persons with disease d compared to persons without disease d, with gender, age, and risk factor classes and states for other diseases being equal. The initial numbers of never and current smokers and the initial disease prevalence rates are calculated from input data:

(5) Nc(0)=nc,0(a(0))Npop,0(a(0))pd(0)=pd,0(a(0))

with: 

a(0) age of cohort at initial time point t = 0

N_c_(t) number of persons in smoking class c at time t,

for c = never, current

N_pop,0_(a) initial total population numbers

p_d_(t) disease d prevalence rates at time t

n_c,0_(a) input proportions of population in smoking classes c

p_d,0_(a) input disease d prevalence rates at age a

The initial values of the numbers of former smokers, stratified by time since cessation, were generated in a pre-processing step by running the model for a birth cohort without any disease included. In this way we calculated the distribution of former smokers n_former_(s,a) over all cessation classes (s), specified by gender and age a. Doing so, we implicitly assumed that all smoking class transition probabilities are constant over time. Thus:

(6) *N*_*former*_(0,*s*) = *n*_*former*,0_(*a*(0))*n*_*former*_(*s*,*a*(0)))*N*_*pop*,0_(*a*(0))

with: 

N_former_(t,s) number of former smokers at time t in former smoking class s

s index over classes for time since smoking cessation, s = 1,...,S

E.g., s = 2 means former smoker stopped 1–2 years ago.

n_former,0_(a) data proportion of former smokers in the population at age a

### Simulation component

The simulation component describes the changes of the prevalence numbers in all smoking classes distinguished, as well as the changes of the prevalence rates for all chronic diseases included in the model. These changes are formulated as differential equations with 1-year time steps.

#### 1-year changes of smoking class prevalence numbers

The mortality rates for all smoking classes at time t (*μ*_c_(t)) depend on the disease prevalence rates. The disease prevalence rates for each smoking class are found by distributing the prevalent disease cases at time t over all smoking classes using relative risks and the smoking class distribution at time t. To do so, first the mean relative risk values are computed for time t.

(7) $$E(RR_{d}(former),t)=\frac{\sum_{s}RR_{d}(s,a(t))N_{former}(t,s)}{\sum_{s}N_{former}(t,s)}$$

(8) $$E(RR_{d},t)=\frac{\sum_{c}RR_{d}(c,a(t))N_{c}(t)}{\sum_{s}N_{c}(t)}$$

with:

t time parameter, with 1-year steps

RR_d_(s,a) disease d relative risk of former smoking class s

at age a, see Appendix 3

E(RR_d_(former),t) mean disease d relative risk value at time t for former

smokers, at time t

E(RR_d_,t) mean disease d relative risk value in entire population at time t

Note that the relative risks of never and current smokers (RR_d_(c,a), c = never,current) are constant values, whilte those of former smokers (E(RR_d_(former,t))) depend on the distribution over all time since cessation classes and thus are re-calculated each year. We assumed for each disease that the distributions of the prevalent and incident disease cases over the smoking classes are equal. Adding mortality from other causes results in the total mortality rates for each smoking class at time t.

(9) $$\mu_{c}(t)=\mu_{other}(a(t))+\frac{\sum_{d}RR_{d}(c,a(t))am_{d}(a(t))p_{d}(t)}{E(RR_{d},t)}$$

with *μ*_c_(t) all cause mortality rates for smoking class c at time t

These mortality rates are transformed to 1-year mortality probabilities assuming constant rate values over the year. Using these mortality probabilities, still denoted as *μ*_c_(t), the following equations describe the 1-year change of the prevalence numbers of never and current smokers (N_c_(t)), and of former smokers specified by time since smoking cessation (N_fomer_(t,s)).

(10) $$\begin{array}{l} N_{never}(t+1)=(1-\lambda_{start}(a(t))-\mu_{never}(t))N_{never}(t)\\ N_{current}(t+1)=(1-\lambda_{stop}(a(t))-\mu_{current}(t))N_{current}(t)+\lambda_{start}(a(t))N_{never}(t)+\sum_{s}\lambda_{relapse}(s)N_{former}(t,s)\\ N_{former}(t+1,1)=\lambda_{stop}(a(t))(1-\mu_{former}(t))N_{current}(t)\\ N_{former}(t+1,s+1)=(1-\lambda_{relapse}(s)-\mu_{former}(t))N_{former}(t,s) \end{array}$$

with 

*λ*_start_(a), *λ*_stop_(a) 1-year start and stop smoking probabilities respectively,

at age a

*λ*_relapse_(s) smoking relapse probabilities that depend on time since cessation class s, see appendix 2

The number of former smokers in the last class S at the end of the year are the sum of the numbers in the last and second last class at the start of the year that do not relapse or die.

#### 1-year changes in disease prevalence rates

We describe the 1-year change in prevalence rates instead of numbers for each disease included. Since the mortality rates for other causes of death are assumed equal for persons with and without the disease, the change in the rate values depends only on the disease incidence and disease related excess mortality rates. The current disease incidence rates are the baseline disease incidence rates times the current mean relative risk value.

(11) *i*_*d*_(*t*) = *E*(*RR*_*d*_,*t*)*i*_*d*,*base*_(*a*(*t*))

with:

i_d_(t) disease d incidence rate at time t

i_d,base_(a) baseline disease d incidence rate at age a

1-year event incidence probabilities were calculated from the rate values assuming constant values over the year. These probabilities are still denoted as i_d_(t). The prevalence rates change as a result of incidence and mortality. This equation is known as the DisMod-equation [28].

(12) *p*_*d*_(*t *+ 1) = *p*_*d*_(*t*) + *i*_*d*_(*t*)(1 - *p*_*d*_(*t*)) - *em*_*d*_(*a*(*t*))*p*_*d*_(*t*)(1 - *p*_*d*_(*t*))

with: em_d_(a) disease d related excess mortality probability at age a.

The parameter em_d_(a) is the excess mortality related to disease d. It describes the additional mortality in the population with disease d as compared to the population without disease d (see the online appendix of [22]). The parameter am_d_(a), that was defined before, describes the additional mortality rate, conditional on all risk factors and other disease states. The parameter am_d_(a) can be interpreted as the mortality that uniquely can be attributed to disease d. It adjusts the excess mortality for mortality due to co-morbid diseases. The part (1 - p_d_(t)) in the last term of the equation comes from describing changes of prevalence rates instead of numbers.

### Model post-processing component

The model output variables are computed from the results in the simulation component. These are the following:

Disease incidence numbers

(13) $$I_{d}(t)=i_{d}(t)\sum_{c}N_{c}(t)$$

QALYs generated (accumulated)

(14) $$QALY=\sum_{t}N_{c}(t)\prod_{d}\left(1-w_{d}(a(t))p_{d}(t)\right)$$

with: 

I_d_(t) disease d incidence numbers during 1-year period [t,t+1)

QALY quality-adjusted life years

w_d_(a) disease d weight coefficients at age a

The weight coefficients w_d_(a) describe the relative loss of quality of life value due to the disease. A value 0 means there is no loss of quality of life due to the disease; a value 1 indicates there is complete loss of quality of life, and that having the disease is no better than being dead.

## Appendix 2: Relapse rates that depend on time since smoking cessation

First we present the statistical regression model that describes how relapse rates depend on the time since smoking cessation. Second, we describe how the regression parameters were estimated based on data available from a retrospective study on smoking behavior. Third, the resulting parameter estimates are presented.

### Statistical model

The relapse rates are the rates at which former smokers restart smoking. They depend on the time since smoking cessation. Our formal model is defined in continuous time. Subsequently, the 1-year probabilities of relapse used in our simulation model were calculated by integrating the rate values over time. Following the literature [1] we assumed that the proportional decrease of these relapse rates is constant over time, meaning that the relapse rates are highest shortly after cessation, and diminish in the long run. As a result of this assumption, the relapse rates follow a negative-exponential curve.

(15) *λ*_*relapse*_(*s*) = *αβ *exp(- *βs*)

with: 

s time since smoking cessation [months]

*λ*_relapse _smoking relapse rate

*α*, *β *regression coefficients.

The parameter *β *(unit: [month]^-1^) governs how fast the relapse rates decline with time since smoking cessation. The parameter *α *governs the lifetime probability of no relapse. The lifetime probability of no relapse depends on the relapse rates accumulated over the entire lifespan. Thus:

(16) S(∞|α,β)=lim⁡t→∞S(t|α,β)=lim⁡t→∞exp⁡(−∫0tλrelapse(s)∂s)=lim⁡t→∞exp⁡(−α(1−exp⁡(−βt)))=exp⁡(−α)

with S(t|*α*,*β*) the probability of no relapse until time t. This result shows that quitters have a positive probability of never relapsing. Since our model uses 1-year time-steps we did not use relapse rates but 1-year probabilities of relapse. These probability values are calculated as integrals over 1-year time periods. For example, the probability of relapse in class s_1_, i.e. between the time points s_1 _and s_1_+1 conditional on no relapse until time s_1_, is:

(17) $$\frac{S(s_{1}|\alpha ,\beta )-S(s_{1}+1|\alpha ,\beta )}{S(s_{1}|\alpha ,\beta )}=1-\exp(-\alpha (\exp(-\beta s_{1})-\exp(-\beta (s_{1}+1)))$$

### Estimation procedure

The parameters *α *and *β *were estimated from a series of cross-sectional population surveys on smoking behavior. The samples were representative for the Dutch population aged 15 years and above [2,33]. Data were collected in the years 2000–2003. The complete questionnaire used is reproduced in Cappanesi *et al.*[32], and includes questions regarding current smoking status as well as past smoking behavior of the responders. These data were used to estimate the model parameters *α *and *β *in the following way.

Firstly, we calculated the numbers of quitters. The 1-year quit probabilities were calculated from the survey by dividing the number of current non-smokers that smoked 1 year ago by the total number of smokers 1 year ago, based on answers in the retrospective survey. These probabilities were multiplied with the proportion of current smokers available from the survey and the total population numbers available from Statistics Netherlands [24] to get the numbers of quitters for each age class.

(18) *N*_*stop*_(*a*) = *λ*_*stop*_(*a*)*p*_*current*_(*a*)*N*(*a*)

with: 

a age

N_stop_(a) 1-year number of quitters at age a

p_current_(a) proportion current smokers at age a

N(a) total population numbers at age a

*λ*_stop_(a) 1-year quit probability of current smokers

Given trial values *α *and *β*, we calculated for each group of quitters specified by age at cessation how many quitters were still abstinent over time using the probability of relapse function.

(19) *N*_*former*_(*a*,*d*|*α*,*β*) = *N*_*stop*_(*a *- *d*)*S*(*d*|*α*,*β*)

with: N_former_(a,d|*α*,*β*) number of persisting former smokers at age a that

quitted d months ago

The notation shows that the calculated number of non-relapsing former smokers depends on the parameters *α *and *β*. This dependency will also be shown for all variables to follow. As a result, for each age class the distribution of all former smokers over time since smoking cessation was calculated using the absolute numbers of all quitters that are abstinent until that age.

(20) $$p_{calc}(a,d|\alpha ,\beta )=\frac{N_{former}(a,d|\alpha ,\beta )}{\sum_{s}N_{former}(a,s|\alpha ,\beta )}$$

with: 

p_calc_(a,d|*α*,*β*) calculated proportion of former smokers at age a who

quitted d months ago

Then we calculated a weighted sum of squares to describe the fit of the calculated proportions to the empirical proportions. The terms of this weighted sum are the squared differences between the expected proportions of former smokers that quitted d months ago from formula 20 and the same proporiton calculated from the data, weighted by the total numbers of former smokers in the ageclass concerned. We aggregated these terms over all classes and over age.

(21) $$SS(\alpha ,\beta )=\sum_{a}N_{former}(a)\sum_{d}(p_{calc}(a,d|\alpha ,\beta )-p_{emp}(d|a){)}^{2}$$

with: 

p_emp_(d|a) the empirical distribution of all former smokers at age a over

time since cessation classes d

SS(*α*,*β*) weighted sum of squares

The empirical distribution of all former smokers p_emp_(d|a) was available from the cross-sectional population survey on smoking behaviour [2]. We used a grid search to minimize the sum of squares. The resulting parameter values *α *and *β *are called the estimated values calculated by the method of weighted least squares.

(22) $$\hat{\alpha },\hat{\beta }=\arg\min_{\alpha ,\beta }SS(\alpha ,\beta )$$

At first, we estimated the parameters *α *and *β *for several age classes separately to check for different declines of smoking relapse rates over age. However, since the number of former smokers was too small for lower and middle ages, we concluded that we could not identify any effect of age, and thus assumed parameter values that were different between both sexes but constant over age.

### Results

Estimated parameter values for $\hat{\alpha }$ and $\hat{\beta }$ for men are 1.177 and 0.150. Estimated parameter values for $\hat{\alpha }$ and $\hat{\beta }$ for women are 1.197 and 0.113.

## Appendix 3: Relative risks that depend on time since smoking cessation

In this appendix the methods used to calculate relative risks of former smokers that depend on time since smoking cessation are described. Two different methods were used to estimate the regression parameters, depending on the data available. The first method was used for diseases with sufficient data on relative risks of former smokers specified by time since cessation. The second method was used when these data were not available. In the latter case we estimated the regression parameter from the distribution of all former smokers over time since cessation (see Appendix 1) and mean relative risks of all former smokers.

### Statistical model

The statistical model is defined for the relative risks of former smokers compared to never smokers as a function of the time since smoking cessation. These relative risks comprise both all cause mortality and incidence of chronic diseases. The relative risks of former smokers decrease over time since cessation, meaning that the effect of past smoking behavior gradually disappears. We made the following assumptions:

- The relative risk of quitters equals the relative risk of current smokers.

- The relative risk of former smokers approaches the relative risk of never smokers, i.e. value 1.

- Relative risks of former smokers show a time-constant proportional decrease.

- The proportionality coefficients that describe the rate of decrease over time of the relative risks decrease proportionally over age

These assumptions result in the following formulas for the relative risk:

(23) $$\begin{array}{l} RR_{former}(a,s)=1+(RR_{current}(a)-1)\exp(-\gamma (a)s)\\ \gamma (a)=\gamma_{0}\exp(-\eta a*(a)) \end{array}$$

with: 

a age

a*(a) transformation of a, a*(a) = (a-50)^+^: the non-negative value of a-50

s time since smoking cessation

*γ *regression coefficient of time dependency

*η *regression coefficient of age dependency

RR_current_(a) relative risks of current smokers at age a

RR_former_(a) mean relative risks of all former smokers at age a

The parameter *γ*_0 _is the reference value of parameter *γ*, i.e. the value of *γ *for age 50 years. We chose age 50 years as the origin, since we could not identify any age gradient for lower ages (see below). As a consequence, we assumed age-constant rates of decrease over time for these ages. Thus, in the statistical model applied in our simulation model the term (a-50) was replaced by the term (a-50)^+^.

### Estimation of regression parameters from cohort studies stratified by time since cessation

To estimate the regression coefficients *γ *and *η *for each smoking-related relative risk, we used data from major cohort studies presented in literature [6-8,35-42]. To be included, studies had to present relative risks that were adjusted for at least gender and age, and the time since smoking cessation had to be subdivided in at least three classes with reported cut-off points. We estimated the regression coefficient *γ *by the method of least squares. For each study we checked whether the published relative risk of current smokers matched the extrapolated one based on the curve of relative risks of former smokers. If so, we added this relative risk value (with time since smoking cessation 0) to the reported ones for former smokers, and re-estimated the rate of proportional decrease. We included these relative risks of current smokers to improve the precision of our estimation. Only for some forms of cancer among which lung cancer we found differences between reported and extrapolated relative risks of current smokers. These differences point at a reversal of causality: getting lung cancer is a reason to quit smoking. We plotted the calculated relative risk curves and data points regressed on the time since smoking cessation to check the model fit for each study separately.

Next, all regression coefficients *γ *calculated were plotted regressed on age to check the assumption of age-constant proportional decrease. Our state-transition model uses 1-year time-steps, whilte most cohort studies have much longer follow-up time periods. Thus, in the cohort studies the age at baseline is not the age at event. Therefore we used the estimated age at event for age a in the formula on *γ*(a) instead of the reported age at baseline. The age at event was calculated assuming a Gompertz-type event rate. I.e., we assumed that the event rate *λ *increases exponentially with age a, i.e. *λ*(a) ∝ exp(a c). We assumed that the rate of increase of all event rates with age was equal to the one for all cause mortality. We estimated this rate value using Dutch mortality data that were available from Statistics Netherlands.

If we found an age-dependent proportional decrease of the rate of change of relative risks with time since cessation, we estimated the regression coefficient *η *by the method of weighted least squares. For ages lower than 50 years we had not enough data to identify any relation with age, and thus assumed constant values of parameter *γ*. Only for all cause mortality we had sufficient data to identify the model parameters specified by age. Based on the data we could find no differences between men and women.

### Estimation of regression parameters from one relative risk value for all former smokers

If no data were available from cohort studies we estimated the regression parameters from reported relative risk values of all former smokers together, and used the calculated distribution of all former smokers over time since cessation (see appendix 1). The parameters were estimated by the method of weighted least squares, where the weights were the number of former smokers for each age class.

Using formula (21) we can write the mean relative risk value of all former smokers as a function of the parameters *γ*_0 _and *η *of the regression model. We simplified the formula by taking the first order approximation of the exponential function.

(24) RRformer(a)=∑s(1+(RRcurrent(a)−1)exp⁡(−γ0exp⁡(−ηa*(a)s))nformer(s|a)≈1+(RRcurrent(a)−1)∑s(1−γ0(1−ηa*(a))s)nformer(s|a)=1+(RRcurrent(a)−1)(1−γ0Eformer(s|a)+γ0ηa*(a)Eformer(s|a))

with: E_former_(s|a): the mean time since cessation of all former smokers at age a. This equation results in an equation on the two parameters *γ*_0 _and *η*_0_:

(25) $$\gamma_{0}E_{former}(s|a)-\eta_{0}a*(a)E_{former}(s|a)=\frac{RR_{current}(a)-RR_{former}(a)}{RR_{current}(a)-1}$$

with: 

*η*_0 _transformed parameter value; *η*_0 _= *η γ*_0_

RR_former_(a) the data mean relative risk value of all former smokers

The latter equation was solved within the framework of weighted linear regression:

(26) (*γ*_0_, *η*_0_) = (*XWX*^*T*^)^-1^*X*^*T*^*Wy*

with: X design matrix, i.e. with as columns the independent variables E_former_(s|a)

and a*(a) E_former_(s|a) for (rows) all age classes

W diagonal weight matrix, with values n_former,0_(a) N_pop,0_(a), the number of

former smokers for all age classes

y response vector, i.e. with as elements the empirical values of the right hand side of equation (25).

We assumed that the decline of the relative risks of former smokers decreased with age. As a result, if equation (24) resulted in a negative value of parameter *η*_0 _we re-estimated parameter *γ*_0 _with fixed value *η*_0 _= 0.

### Results

Table 2 displays estimations of parameters *γ*_0 _and *η *for all 14 smoking related diseases distinguished in the CDM.

**Table 2:** Results of parameter estimates per smoking related disease

	*γ*_0_	*η*
AMI	0.24228	0.05822
Other CHD	0.24228	0.05822
CHF*	0.0421371	0
CVA	0.31947	0.01648
COPD	0.20333	0.03087
Diabetes*	0.024811	0
Lung cancer	0.15637	0.02065
Stomach cancer*	0.0264112	0
Esophagus cancer*	0.0537424	0
Larynx cancer*	0.0279918	0
Uriny/bladder cancer	0.05417	0
Kidney cancer*	0.0385957	0
Pancreas cancer	0.09279	0
Oral cavity cancer*	0.0493028	0

* Estimated using relative risks of all former smokers combined and using the calculated distribution of all former smokers over time since cessation (see Appendix 3).

## Competing interests

The author(s) declare that they have no competing interests.

## Authors' contributions

RTH developed the simulation model and PHMvB carried out the analyses. All authors contributed to the writing of the manuscript.
